# Comparison of Artificial Neural Network and Polynomial Approximation Models for Reflectance Spectra Reconstruction

**DOI:** 10.3390/s23021000

**Published:** 2023-01-15

**Authors:** Mihael Lazar, Aleš Hladnik

**Affiliations:** Department of Textiles, Graphic Arts and Design, Faculty of Natural Sciences and Engineering, University of Ljubljana, Snežniška Ulica 5, SI-1000 Ljubljana, Slovenia

**Keywords:** reflectance reconstruction, artificial neural network, polynomial approximation

## Abstract

Knowledge of surface reflection of an object is essential in many technological fields, including graphics and cultural heritage. Compared to direct multi- or hyper-spectral capturing approaches, commercial RGB cameras allow for a high resolution and fast acquisition, so the idea of mapping this information into a reflectance spectrum (RS) is promising. This study compared two modelling approaches based on a training set of RGB-reflectance pairs, one implementing artificial neural networks (ANN) and the other one using multivariate polynomial approximation (PA). The effect of various parameters was investigated: the ANN learning algorithm—standard backpropagation (BP) or Levenberg-Marquardt (LM), the number of hidden layers (HLs) and neurons, the degree of multivariate polynomials in PA, the number of inputs, and the training set size on both models. In the two-layer ANN with significantly fewer inputs than outputs, a better MSE performance was found where the number of neurons in the first HL was smaller than in the second one. For ANNs with one and two HLs with the same number of neurons in the first layer, the RS reconstruction performance depends on the choice of BP or LM learning algorithm. RS reconstruction methods based on ANN and PA are comparable, but the ANN models’ better fine-tuning capabilities enable, under realistic constraints, finding ANNs that outperform PA models. A profiling approach was proposed to determine the initial number of neurons in HLs—the search centre of ANN models for different training set sizes.

## 1. Introduction

Colour information about objects, such as works of art or graphic materials, is usually captured using a camera or a scanner. RGB colour readings of the same surface obtained with different cameras often differ markedly, even when the illumination is unchanged. The acquired colour values depend on the illumination, optical properties, and sensor characteristics of the device. Even the colour information given in device-independent colour spaces such as XYZ or CIELAB incorporates illuminance and characteristics of the observer. In contrast, the spectral values of the object’s surface reflectance provide colour information that is independent of these factors. The hyperspectral information is used in many fields of science and in practical applications, among them agriculture [1,2], remote sensing [3,4], food industry [5], astronomy [6], health care [7], and cultural heritage [8,9]. If the surface is large and uniformly coloured, its reflectance can be measured with a spectrophotometer. In the case of changing colours typically found in works of art, using a spectrophotometer is not possible, but the acquisition of more authentic object colour information can be made in various ways. Multispectral cameras, which describe an image with more than three frequency bands, and hyperspectral cameras, where the image description contains a large number of narrow frequency bands with a wavelength step of 10–20 nm, require special equipment, either sequential exposure in different frequency bands [10], sequential acquisition of an object image through different filters [11], or an expensive multispectral camera [12,13]. Multi/hyperspectral devices are expensive, and their temporal resolution may decrease while increasing spatial and frequency resolution [14]. However, multidimensional reflectance spectra can also be estimated or reconstructed from RGB camera responses, which is an attractive alternative due to the affordability of commercial cameras with high spatial resolution and fast image acquisition. Some of the many methods of spectrum reconstruction from camera readings have already been mentioned in our previous work [15,16], and some are described below.

Slavuj and Green [17] have performed a reflectance reconstruction from RGB values for different materials and printing techniques based on a significant dimensionality reduction of the measured reflectances through the use of principal components analysis (PCA) by knowing the response of the camera sensor through characterisation with monochromatic light and a concise control of scene illumination. The reconstructed reflectances were obtained mathematically through the matrix inversion process. The authors concluded that the results were heavily influenced by media surface characteristics, and the resulting reconstruction error could be reduced by optimising the camera sensitivity characterisation, selecting appropriate colour samples and material surfaces and increasing/optimising the number of principal components. Cao, Liao, and Cheng [18] proposed the reflectance reconstruction of camera RGB readouts by weighing the known reflectances of selected training samples. Four weighing modes were presented, and the influence of a different number of nearest training samples was investigated. As the method assembles unknown reflectance values from samples with known reflectances captured in the same camera and lighting conditions, no prior knowledge about the camera’s spectral responsivity is required. Oh et al. [19] reconstructed the reflectance spectra and illumination by iteratively solving matrix equations, using RGB responses from three cameras with known sensor sensitivities, and assuming an efficient dimensionality reduction of the real-world material reflectance and real-world illumination. In recent years, the use of convolutional neural networks (CNN) has come to the fore with the increasing power of CPUs and GPUs. Zhao et al. [20] consider that the high cost of the hyperspectral imaging device and the lower resolution of the hyperspectral images are limiting factors for broader applications in agriculture and other fields. They suggest performing a reconstruction of the spectrum from RGB photos captured by mobile phone using a residual neural network model named HSCNN-R, trained from hyperspectral camera data and RGB images computed from these hyperspectral data. Special attention was paid to the selection of loss function and evaluation metrics. The proposed system predicts the fruit quality from the reconstructed spectral images using random forest regression. Stiebel et al. [21] utilised a U-Net architecture of a CNN to map RGB to spectral image based on not only individual camera signals but also making use of the neighbouring local contextual information. The training was made possible by using the largest hyper-spectral data set available to that date, with about three hours of GPU time for training an individual network. Zhao et al. [22] presented multispectral image (MSI) reconstruction models to support the application for plant phenotyping in precision agriculture. The natural colour RGB (ncRGB) images were first generated from the benchmark MSI dataset. Then, ncRGB to MSI reconstruction model was trained with a prepared training set of ncRGB–MSI image pairs integrating different loss functions, of which the mean relative absolute error (MRAEloss) and spectral information divergence (SIDloss) appeared to be the most effective, with models using the former being more robust towards variability between growing seasons and species. The resulting MSI images enable higher capabilities in differentiating the three levels of irrigation treatments of maize plants. However, despite extensive research based on CNNs, for smaller amounts of data, multilayer perceptron or plain fully connected neural networks are the appropriate choice. Gong et al. [23] consider that compared to deep networks (such as deep CNNs) built for large datasets, the use of a backpropagation neural network (BPNN) is more advantageous in small datasets. In the study, a significant improvement is achieved in chlorophyll prediction with the proposed BPNN in comparison to the pseudo-inverse method of spectral reflectance reconstruction from RGB images. The neural network training was performed with the dataset of hyperspectral camera acquisition of the green leaves and the RGB photos taken by smartphone in the same position as the hyperspectral device. Lopez-Lopez and Mendez-Rial [24] observed the colourimetric and reflectance reconstruction performance of the simple linear model and multilayer perceptron (MLP) using ReLu activation function and mean squared error loss function from input data of both RGB and hyperspectral snapshot cameras. The MLP approach with RGB data yielded the best results because of the narrower band of hyperspectral camera spectral sensitivity. They conclude that due to large spatial resolution, the MLP approach with the snapshot camera data appears as a viable and interesting solution to automate quality control in manufacturing processes.

Methods based on mathematical models usually require knowledge of image acquisition settings and conditions, such as illumination conditions, camera characteristics, and sensory response. However, consumer camera manufacturers do not always provide this information, and lighting information is often unavailable. In the presented research, we study the possibility of converting RGB responses to the reflectance frequency spectrum with the help of object-parallel acquisition of reference colour samples with known spectral values and the subsequent use of a reflectance reconstruction model trained from this learning set of reference samples. In our previous study [16] we demonstrated the possibility of using an artificial neural network (ANN) model with one hidden layer (hereafter 1HL) of neurons and a learning set (hereafter LS) composed of readings from one or more different cameras, whose parallel channels must be linearly independent for an efficient reflectance reconstruction. The question remains whether increasing the number of hidden layers (hereafter HLs) can lead to a further improvement in the ANN-based reflectance reconstruction performance, which could already be expected by adding at least one more HL. The use of simple (“vanilla”) ANNs is attractive because the modelling is relatively straightforward from a software point of view, compared to CNNs, and the performance of the resulting ANN is fast when run on a CPU and an order of magnitude faster when using a GPU.

Data of the reflectance spectrum (hereafter RS) can be used in various ways, e.g., with subsequent choice of different lighting options (e.g., with the standard illuminants, such as incandescent, daylight, or fluorescent lighting), colour reconstruction is possible, e.g., in XYZ, CIELAB, or sRGB for presenting the observed objects (artistic paintings) in such lighting conditions. Depending on the light reflection, the surface type is essential, as the reflection can be specular (regular) or diffuse. In our future work, we intend to capture the colours of works of art in pastel, chalk, crayons, tempera, and watercolour, as well as prints on matte paper as closely as possible, so in our experiment, only non-glossy (matte) samples were used.

## 2. Reflectance Reconstruction

Our idea is to build a system that would map a smaller number of input values—RGB responses of one or more cameras (1/2/3 RGB)—into a larger number of reflectance spectra output values. The functional connection between system inputs and each output value (spectrum component) can be implemented in different ways, e.g., via a single- or multilayer ANN, or using a multivariate polynomial approximation (PA) approach. For all our investigated colour samples, which mainly include natural colours, their reflectances are smooth continuous functions. It was demonstrated by Hornik [25] that multilayer feedforward networks are, under very general conditions on the hidden unit activation function (continuous, bounded, and nonconstant), universal approximators provided that sufficiently many hidden units are available. The single-layer ANN RS reconstruction performance has also been explored in our previous experiment, whereas the question of possible efficiency improvement by adding HLs of neurons remained open, where an upgrade could be expected already with one additional HL. The study of Rolnick and Tegmark from MIT [26] was focused on the ability to approximate natural functions expressed by multivariate polynomials with single- and multilayer ANNs, where the number of neurons *m* with respect to the number of input variables *n* in a single-layer ANN grows exponentially with *n*, and in a multilayer ANN exponentially with *n^1/k^* (where k is the number of layers), i.e., in the case of two layers it grows with the *square root of n*. Since in our study we had three, six, or nine input variables with one, two, or three cameras, we intended to—considering realistic limitations, especially the limited size of the LS and the number of input variables—compare the RS reconstruction performance of one and two-layer fully connected neural networks based on a large-scale numerical experiment and then compare these with the RS reconstruction performance of models based on multivariate polynomials, which were also affected by the aforementioned realistic limitations.

### 2.1. ANN Modelling

The use of neural networks has boomed in the last decade with the availability of multi-core graphics cards with massively parallel processing, and at its forefront are convolutional and deep neural networks, which can learn from large amounts of input data and absorb much knowledge. In our case, when there are only a few hundred, up to a thousand, input–output pairs in the LS, there is no need to reduce the input amount of data to find the best possible functional connection between them. Therefore, our system was modelled as a fully connected neural network, which receives readings from one to three cameras, followed by the first HL (hereafter HL1) of neurons, then the second HL (hereafter HL2)—in the case of a two-layer network, and an output layer with 36 outputs of real values, which make up vector of reconstructed RS—spectral power distribution of visible light from 380 to 730 nm in 10 nm wavelength steps.

**Figure 1:**
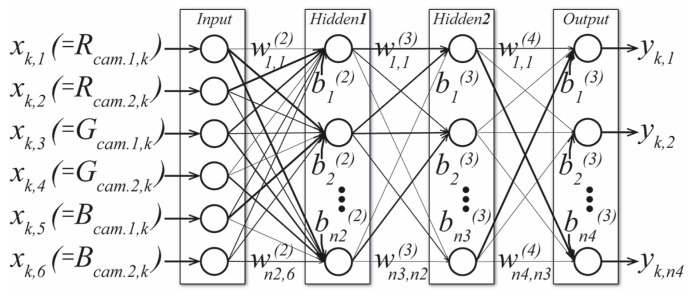
The architecture of our ANN with two hidden layers and a variable number of hidden neurons, employed for the reconstruction of reflectance spectral components from 1 to 3 camera RGB responses.

The generic architecture of the employed ANN, depicted for two HLs (hereafter 2HL) is shown in Figure 1, where *x_k,j_* is *j*-th ANN input of *k*-th R/G/B response from a selected number of cameras (depicted for two); *b_j_*^(*L*)^ and *w_j,i_*^(*L*)^ stand for biases and connection weights, and *y_k,j_* are output layer neurons’ output values, representing 36 spectral components of *k*-th reconstructed reflectance; *j* is neuron index in (*L*)-th layer and *i* in (*L*-1)-th layer.

Having LS of (1/2/3 RGB, reflectance spectrum) pairs from the reference set of patches, the training of the ANN is performed by a learning algorithm, adjusting connection weights and biases so that the network cost function—the *mean squared error* (hereafter MSE)—is minimised (Equation (1)).

(1) $$C=\frac{1}{N_{p}}\sum_{k=1}^{N_{p}}\left[\frac{1}{N_{\lambda }}\sum_{j=1}^{N_{\lambda }}{\left(r_{k,j}-y_{k,j}\right)}^{2}\right],$$

*N_p_* is the number of patches, *N_l_* is the number of reflectance wavelengths, *k* is patch index, and *j* wavelength index; *r_k,j_* is a *j*-th spectral component of *k*-th patch *measured* reflectance and *y_k,j_ j*-th spectral component of *k*-th patch *reconstructed* reflectance.

The programming and testing of ANNs for our experiment were done with the Matlab software package, including the Deep Learning Toolbox module, where two learning algorithms were implemented for the function approximation. “Standard Backpropagation-Based Gradient Descent” (hereafter BP) is fast as it can be executed on a GPU, while the other one, the Levenberg–Marquardt algorithm (hereafter LM), can only run on a CPU, but is specially adapted to minimise the sum-of-squares error functions [27]. Even though in our previous research it was shown that the LM learning algorithm requires at least an order of magnitude more time, the present experiment was performed in parallel with both algorithms since the LM algorithm has an order of magnitude better learning convergence by means of epoch repetition and better ANN performance compared to BP learning algorithm.

### 2.2. Polynomial Approximation Modelling

**Figure 2:**
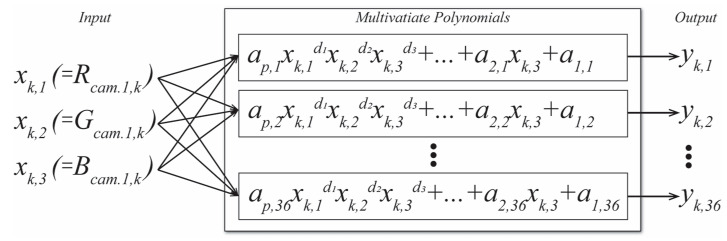
Reflectance reconstruction using polynomial approximation models.

The PA reflectance reconstruction model is designed as a collection of multivariate polynomials, each of which models one of the reflectance components (Figure 2). The independent variables *x_k,i_* of each polynomial are the *i*-th inputs, R, G, and B responses of one, two, or three cameras (depicted is a one-camera model), and each calculated value *y_k,j_* is the *j*-th output, individual component of the *k*-th patch reflectance spectrum. An example of a multivariate polynomial of the 2nd degree to calculate the *j*th spectral component of the kth colour patch from one-camera responses is shown in Equation (2),

(2) $$y_{k,j}=a_{10,j}x_{k,1}^{2}+a_{9,j}x_{k,2}^{2}+a_{8,j}x_{k,3}^{2}+a_{7,j}x_{k,1}x_{k,2}+a_{6,j}x_{k,1}x_{k,3}+a_{5,j}x_{k,2}x_{k,3}\;+a_{4,j}x_{k,1}+a_{3,j}x_{k,2}+a_{2,j}x_{k,3}++a_{1,j},$$

where *x*_*k*,1_, *x*_*k*,2_, and *x*_*k*,3_ are R, G, and B one-camera responses of the *k*th colour patch and *a_m,j_* are polynomial coefficients. The number of multivariate polynomial coefficients *p* increases according to Equation (3) [28],

(3) $$p=\left(\begin{array}{c} n+d\\ d \end{array}\right)=\frac{\left(n+d\right)!}{d!\;n!},$$

where *n* is the number of independent variables, and *d* is the degree of the polynomial. It must hold that $d\geq d_{1}+d_{2}+\cdots +d_{i}$, where *d_k_* are the exponents of independent variables at each term of the polynomial.

The model with the best performance was recorded for every combination of cameras, a training set size, and polynomial degree. For the PA model efficiency estimation, MSE—the same measure as the ANN cost function—was used. In PA modelling, with a gradual increase in the degree (starting at 1) of the multivariate polynomial, the reconstruction efficiency at first improves, then decreases. If the degree of the polynomial is further increased, the error becomes unacceptably large, or the algorithm (Matlab Polyfitn [29]) starts reporting warnings referring to the possibility of an unsuccessful model due to insufficient data for calculating the multivariate polynomial of a higher degree. The PA model with the multivariate polynomials of the highest possible calculated degree is not the best in any of the calculated cases. It turned out that the best PA models reside mainly in the middle between the 1st and maximum possible degree of the polynomial that can still be modelled with an available amount of data.

### 2.3. Hypotheses

Our hypotheses are as follows:

**Hypothesis** **1.** *In two-layer ANNs with a smaller number of inputs (1/2/3 RGB) and a larger number of outputs (RS), ANNs with a larger number of neurons in the second than in the first HL will perform better.*

**Hypothesis** **2.** *Reflection reconstruction performance is better using ANN with two HLs than ANN with a single HL of neurons.*

**Hypothesis** **3.** *Increasing the training set at a constant number of cameras improves the RS reconstruction performance of both ANN and polynomial models.*

**Hypothesis** **4.** *For a constant size of the training set, increasing the number of cameras improves the RS reconstruction performance of ANN compared to polynomial models due to the rapid increase in the number of coefficients in the latter.*

**Hypothesis** **5.** *The performance of reflectance spectra reconstruction using PA and ANN models is comparable, but with realistic constraints, such as limited LS size and multi-camera data, it is possible to find better-performing models with ANN than with PA.*

## 3. Materials and Methods

In our previous experiments, it was found that when using an ANN with 1HL of neurons, by increasing the number of cameras, i.e., using two or three RGB responses instead of just one, RS reconstruction performance noticeably improved. The purpose of the current study is to investigate the possibility of further improving the efficiency of ANN models by increasing the number of HLs, where we assume that the improvement could already be detected with just one additional layer of hidden neurons, and then comparing the RS reconstruction efficiency of 1HL and 2HL ANN with PA models. The learning sets are structured in the same way for all observed systems, the inputs are 3, 6, or 9-dimensional vectors of RGB responses, and the outputs are higher-dimensional vectors of reflectance spectra—readings of the spectrophotometer.

The source of colourimetric data for our experiment was The Munsell Book of Color Matte Collection, with 44 sheets providing 1301 colour patches, varying hue, chroma, and value. Forty sheets are divided into 2.5 steps Munsell hue circle (2.5 to 10 for Red, YR, Yellow, GY, Green, …, Purple, and RP), and the four remaining sheets contain subtly hued and neutral greys. The reflectance spectrum of each patch was measured by spectrophotometer X-Rite i1Pro 2 at five points (on both diagonals, a quarter of the distance from each corner, and at the patch centre) with a maximum measuring standard deviation under 0.4%, resulting in five vectors with 107 components for wavelengths from 376.66 to 730 nm (with a step size of 3.33 nm). The extended RS vectors (LS output values) have been calculated as the five-point RS measurement averages.

Our source for LS includes many natural colours (e.g., soil, skin, foliage), and the extended RS vectors are defined in a 3.33 nm wavelength increment. Surface spectral reflectances of many organic and inorganic substances are characteristically smooth, low-pass functions of wavelength [30]. Tristimulus errors are assumed to be negligible using a 5 nm wavelength increment, and because of the absorption characteristics of human-made and natural colourants, the sampling rate can be significantly decreased [31]. The smoothness of reflectance curves has been confirmed by spectrophotometric readings of all measured colour patches in the 3.33 nm step. Therefore, the decrease in the sampling rate of LS is acceptable. Subsampling was made from 3.33 to 10 nm step, resulting in 36 reflectance spectral components in the range from 380 nm to 730 nm.

**Figure 3:**
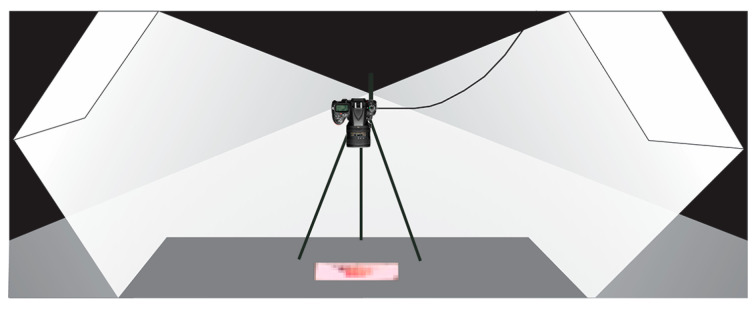
Photo studio setting.

All Munsell Matte colour patch sheets were photographed in a photo studio under constant and uniform lighting conditions. Similarly to the experiment setup described in our recent paper when two cameras were involved [32], in the present study we used three cameras (C1 = *Nikon D600*, C2 = *Nikon D700* and C3 = *Panasonic GH-4*) with disabled automatic settings (Figure 3). The light source spectral power distribution has been measured with a spectrophotometer at the sheet location, with a correlated colour temperature (CCT) of 3019° K. Colour patch sheets were photographed in RAW format and subsequently converted to 8-bit RGB—normalised using Adobe Lightroom software to the measured CCT. The tint was balanced to 0, and chromatic aberration, even though unnoticeable, was corrected with adequate lens profiles. The process resulted in converting digital photos into AdobeRGB (1998) colour space. The RGB value of each patch was calculated as the median (rather than the mean) of the inner 50% of the squared patch area to avoid possible biasing due to minor colour deviations.

The complete set of RGB-reflectance pairs included data of all 1301 Munsell Matte colour patches, with three cameras’ sets of RGB values and 36 values for each RS vector. Before training the ANN and PA models, the complete set was reshaped into a subset according to the observed combination of cameras and then split into a learning set and the remaining set of *independent samples*, for which additional measures of RS reconstruction performance were also calculated. At the beginning of each ANN or PA model training iteration, the LS was randomly split into training, validation, and test sets in a 70:15:15 ratio.

Most of the time, ANN learning algorithm finds a local instead of the global minimum of the cost function. Hence, for each model with selected cameras, training set size, and number of neurons (hereafter NoN) in the HLs, the search for optimal ANN parameters was repeated 41 times. The NoN in ANNs with 1HL varied from 3 to 48 in the step of 1 and with 2HL in the step of 3, resulting in 46 hidden-layer configurations for 1HL and 256 for 2HL ANNs. By probing with 21 ANN training repetitions, it was indicated that increasing the NoN in 1HL and 2HL ANNs above 48 does not improve the ANNs’ RS MSE performance significantly, while the calculations become, especially with LM learning algorithm, extremely time-consuming.

Calculations of ANNs with BP and LM Las and PA models were performed for seven camera combinations, five different learning sets, and corresponding sets of independent samples (Table 1). We tended to increase the possibility of finding the best ANN models, so the number of learning repetitions was almost doubled. The odd number of 41 repetitions was chosen because of some additional statistics not presented in the article.

## 4. Results

In our experiment, the most calculation-intensive was the task of modelling the 2HL ANNs. Systematic experimentation with BP and LM algorithms, seven camera combinations, five LS sizes, and 256 combinations of the first and second hidden-layer NoN with each configuration run 41 times resulted in 734.720 2HL ANN models, with an additional 132.020 1HL ANN and 9184 PA models. Unfortunately, a slower CPU-executed LM learning algorithm shows better results than a significantly faster GPU-executed BP algorithm [15]. The calculations were executed using three computers: a 4-core 2nd gen. i7 CPU with Nvidia 550 GPU, a 6-core 9th gen. i7 with Nvidia RTX 2060 GPU, and an 8-core AMD Ryzen 7 4700U with integrated Radeon Graphics. Calculations consumed more than 8793 h (≈366 days) of CPU and 392 h (≈16 days) of (Nvidia) GPU time.

In the robust grid search for the best models, for each combination of hyperparameters (camera-combination, LS size, and, for ANN, also the learning algorithm, number of HLs, and NoN in each HL), the mean MSE of 41 calculations for each model was calculated, and the model with the best MSE performance was found. RS reconstructions were then calculated for the best models and compared to the original RS. The *Goodness of Fit Coefficient* (hereafter GoFC) and *CIE 2000 colour difference* (hereafter ∆E*_00_) and their averages—Average Goodness of Fit Coefficient (hereafter AGoFC) and Average CIE 2000 colour difference—considering four selected illuminants—A, D50, D65, or F2 (hereafter A∆E*_00_)—were calculated for the test set, independent-set, as well as their weighted sums, according to the number of colour patches in test set and independent set. For each of the 35 camera-combinations/LS combinations, the best performance measures (MSE, AGoFC and A∆E*_00_) and their locations (first/second HL NoN or PA degree) were found.

The analysis of the performed calculations took place in several steps as described in the following subsections.

### 4.1. MSE Performance of 2HL ANN According to NoN in HLs

To determine the influence of the number of neurons in the first and second HLs, *the comparison of the best MSE performance for ANN models with 2HL, where there is a larger NoN in HL1 than in HL2, to the ANN models, where the reverse applies*, has been made.

**Figure 4:**
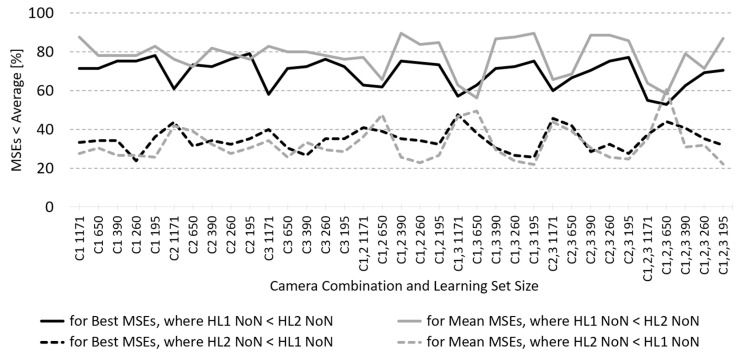
Percentage of best and mean test set MSEs that are lower (better) than their averages in LM-trained ANNs, according to the number of neurons (NoN) in the first and the second hidden layers (HL1, HL2).

For this comparison, a matrix containing the smallest test set MSEs was built for each of the 35 camera-combination/LS size combinations and both learning algorithms. Each row contained MSEs for a selected NoN in HL1 and ascending NoN in HL2, where NoN in both layers (rows and columns of the matrix) increased from 3 to 48 in step 3. Each MSE was compared to the average MSE through all HL1 and HL2 NoN. If the percentage of MSE values above the diagonal of the matrix, which are lower than the average MSE, is higher than below the diagonal, it could be assumed that the best MSEs are smaller for the ANNs with a smaller NoN in the first than in the second HL. The same calculations were also made with the matrices of mean values. The percentage of LM-trained ANN MSEs above and below the diagonal for the best and the mean MSEs through all 35 camera-combination/LS size combinations is shown in Figure 4. The assumption turns out to be true for all but one camera-combination/LS combination. This suggests that for two-layer ANNs, in the rest of the experiment, when studying the properties related to the number of neurons in the HLs, HL1 NoN should be the prime parameter. This part of the experiment also showed that the performance of ANNs with any number of camera inputs and only three neurons in either the first or the second HL is very poor—the MSE is extremely high. The same is true for the three-camera ANN models with six neurons in any layer so these results are not included in this report.

A comparison between the performance of 1HL and 2HL ANN models is possible in several ways. We used two methods in our research. In the first method, models with the same number of neurons in HL1 were compared in parallel within the same camera-combination/LS size/ learning algorithm combination. In the second method, for each camera-combination/LS size/learning algorithm combination, the best performing 1HL and 2HL models, regardless of HL1 or HL2 NoN, were found and then compared with each other and also with the best PA models.

### 4.2. MSE Performance of 1HL and 2HL ANNs with Equal NoN in HL1

To determine the effect of the number of HLs on the performance of ANNs, the *comparison of the best and average MSE performance for ANN models with one and two HLs, according to the HL1 NoN,* was performed.

**Figure 5:**
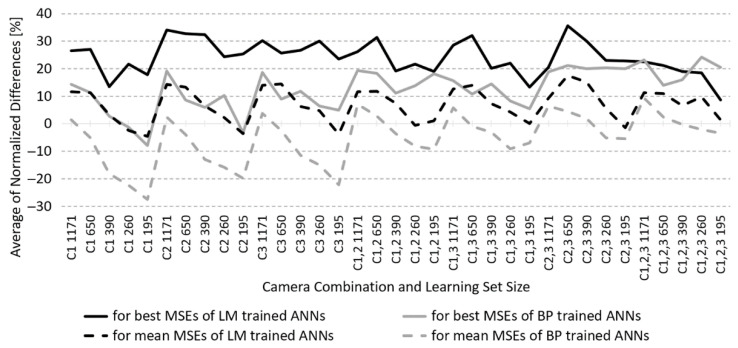
Average of normalised differences between MSE performances of ANNs with one and ANNs with two hidden layers, according to best or mean test set MSEs and using BP or LM training algorithm.

Here, a comparison using the first method is made based on the average of the normalised differences (AoND) of the best and the average MSEs calculated in three steps. First, at each camera-combination/LS size/learning algorithm combination for 2HL ANN models, the search at each NoN of the first HL has been made to find the minimum of the best MSEs through all the values of NoN in the second HL. Then, at each NoN of the first HL, the difference between the 1HL ANN best MSE and the previously found minimum of the 2HL ANN best MSE has been calculated and normalised relative to this minimum. Finally, the average of these normalised differences was calculated through all the HL1 NoN. The AoND for the best and the mean MSE performance is thus calculated and examined for each camera-combination/LS size and learning algorithm. The AoND between best/mean test set MSE performances of BP/LM-trained 1HL and 2HL ANNs is shown in Figure 5.

With BP-modelled ANNs, the AoND for the best MSEs reaches up to 24% in favour of 2HL ANNs. Only with one-camera models and a small LS size did 1HL ANN perform better compared to 2HL ANN. The average AoND through all camera-combination/LS size combinations is small, just above 12%. When the AoND for the mean (instead of the best) MSEs are monitored, it is mostly in favour of 1HL ANN models and is higher for 2HL ANN models only with large LS sizes.

With LM-modelled ANNs, the AoND of the best MSEs ranges between 13% to 35% in favour of 2HL ANN models. Only in models with three cameras and the smallest LS does it drop to around 8%. The average AoND of the best MSEs, through all camera-combinations/LS size combinations, is high, more than 24%. When the mean (instead of the best) MSEs are observed, the AoND reaches 17%, but for one-camera combinations with small LS size, it drops below zero in favour of 1HL ANNs. Still, the average AoND here is higher than 7%.

Next, according to the second comparison method, the best performance obtained for BP- and LM-trained 1HL and 2HL ANNs and for PA models depending on the combination of cameras and LS size will be compared, regardless of HL1/HL2 NoN for ANN and regardless of polynomial degree for PA models.

### 4.3. Influence of LS Size on ANN and PA Model Performace

By varying the LS size, the changes in the best performance of ANN and PA models with a constant number of cameras have been compared.

**Figure 6:**
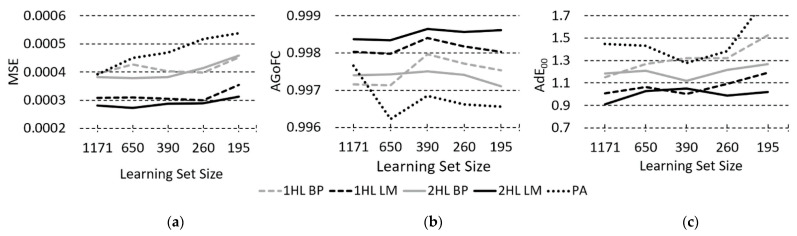
Examples of an in-number-of-cameras average best test set performances: (**a**) MSE for two cameras, (**b**) AGoFC for one camera, and (**c**) A∆E*_00_ (D50 illuminant) for three cameras, all according to the learning set size.

To study performance change trends of the test set MSE, AGoFC and A∆E*_00_ for light sources A, D50, D65, and F2 for one and two cameras, the average performance of three individual cameras and of three two-camera combinations were calculated. For three cameras, averaging is, obviously, unnecessary. Some typical examples of performance change trends (MSE for two, AGoFC for one, and A∆E*_00_ for D50 illuminant for three cameras) according to decreasing LS size are shown in Figure 6.

With the selected *number of cameras*, the MSE of both the ANN and PA models gets slightly worse as the LS decreases. For 1HL and 2HL LM-trained ANN models, the change in MSE through all three number-of-cameras is less than 0.0001; for BP-trained ANN models, it is less than 0.00015; and for PA models, it is greater, between 0.00014 and 0.00034. For all number-of-cameras, the MSEs of the LM-trained ANN models are noticeably lower than those of the models trained with BP. For one camera, the MSEs of PA models are lower than those of BP ANN models, but this is no longer true for two and three cameras.

AGoFC, with decreasing LS, remains constant or improves only slightly for all number-of-cameras in ANN models (for LM ANNs by less than 0.0005, in BP ANN models by less than 0.001), while for PA models, it deteriorates with one and two cameras (0.0014 to 0.0028), and with three cameras it improves a little in the range of up to 0.001. The AGoFC of the PA models is worse than that of the ANN models in almost all cases. It slightly outperforms the BP models only for the largest LS with one and two cameras.

The A∆E*_00_ for all models (ANN and PA) for all illuminations and all number-of-cameras increases with decreasing LS. The change is smaller in LM-trained ANN models (between 0.1 and 0.23), followed by BP ANN (between 0.13 and 0.38), and is larger in PA models (between 0.32 and 0.72).

MSE, AGoFC, and A∆E*_00_ performances for both LM- and BP-trained 2HL ANN models are generally better than those of 1HL ANN models.

### 4.4. Impact of the Number of Cameras on the Performance of ANN and PA Models

By varying the number of cameras, the changes in the best performance of ANN and PA models with a constant LS have been compared.

**Figure 7:**
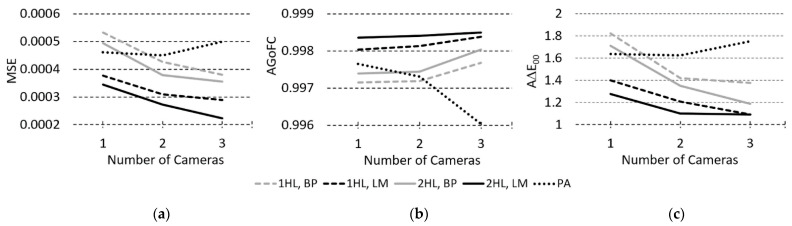
Examples of an in-number-of-cameras average best test set performances: (**a**) MSE for LS size of 650, (**b**) AGoFC for the largest LS size, and (**c**) A∆E*_00_ (F2 illuminant) for the smallest LS size, according to the number of cameras.

In this comparison, just as in the previous one, the average performance values for three individual cameras and three two-camera combinations were calculated. Changes in MSE, GoFC, and ∆E*_00_ performance (for selected illuminants) of ANN and PA models were studied depending on the number of cameras. Some typical examples of performance change (MSE for LS size of 650 samples, AGoFC for the largest LS size of 1171 samples, and A∆E*_00_ with F2 illuminant for the smallest LS size of 195 samples) according to increasing number of cameras are shown in Figure 7.

The MSE improves with increasing number of cameras in all ANN models for the selected constant LS size. For LM-trained ANN models, improvement ranges from 0.00009 to 0.00018; for BP-trained ANN models, it ranges from 0.00013 to 0.00022; whereas for PA models, MSE for three cameras increases (from 0.0004 to 0.00023). For all LS sizes, the MSE of the LM ANN models is lower than that of the BP models. Interestingly, for all LS sizes, the MSE of PA models outperforms the BP-trained ANN models for a single camera but not for the two and three cameras.

GoFC for all five observed sizes of LS with increasing number of cameras remains the same or improves only slightly (by less than 0.0009) for ANN models. For PA models at the largest LS and with increasing number of cameras, GoFC gradually decreases (by −0.0016), while for the other LS sizes, for the three cameras, it improves slightly (by 0.0007), with an intermediate swing at two cameras in the positive or negative direction (−0.002 at the smallest LS). As already noted in the previous comparison, the GoFC for the PA models is worse than that for ANN models in almost all cases; only at the largest LS with one and two cameras it slightly surpasses the BP models.

For all five observed LS sizes, with increasing number of cameras, ∆E*_00_ (for all illuminants) in 1/2HL LM- and 2HL BP-trained ANN models improves (from 0.24 to 0.35). For the 1HL BP-trained ANNs with the smallest LS, ∆E*_00_ performance (for A, D50, and D65 illuminants) with three cameras is slightly worse than with two (up to 0.14) or the improvement is small (Figure 7, right), while for PA models, ∆E*_00_ with three cameras is in the 70% of cases noticeably worse (up to 0.40) than with two cameras.

Again, MSE, GoFC, and ∆E*_00_ performances for both LM- and BP-trained 2HL ANN models are generally better than those for 1HL ANN models.

### 4.5. Comparison of the Best Performances of ANN and PA Models

To find the best models, the sequence of comparisons of ANN and PA models’ best performances according to MSE, AGoFC, and A∆E*_00_ (for all illuminants) was performed.

The performances of ANN and PA models with the best test set MSE are compared below. For each combination of camera-combinations, LS size, and BP/LM learning algorithm, we have 46 models for 1HL ANN (for HL1 NoN from 3 to 48), for 2HL 256 models (for HL1 and HL2 from 3 to 48 in a step of 3), and for PA a different number of the models with the best MSE, according to the highest possible calculated degree of the polynomial.

**Figure 8:**
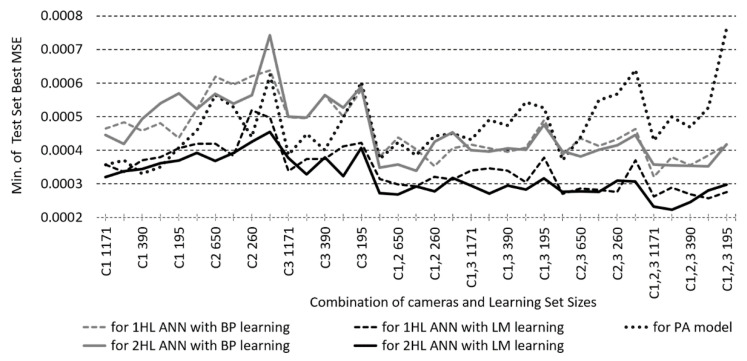
Minimum of Test Set Best MSEs searched through all first and second hidden layer number of neurons combinations, according to one and two hidden layer ANNs (1/2HL) modelled with BP or LM learning algorithm and for the polynomial approximation models (PA).

To compare the best test set MSE for each camera-combination/LS size/learning algorithm combination, first, the models with the smallest MSEs have been found: for 1HL and 2HL ANN by searching through all HL1/HL2 NoN combinations, and for PA models through all available degrees of polynomials. Then, the smallest MSEs for ANN and PA were compared for all camera-combination/LS size combinations (Figure 8). The comparison shows that the MSE performance of LM-trained ANN models is better than both BP-trained ANNs and PA models. For BP ANNs, the best MSE of 1HL and 2HL models overlaps, while for LM ANNs, the best MSE of 2HL models is slightly better than that of 1HL models. The best MSE of the PA models outperforms the BP-trained ANN models with one camera but performs poorer than BP-trained ANN models with two and three cameras.

**Figure 9:**
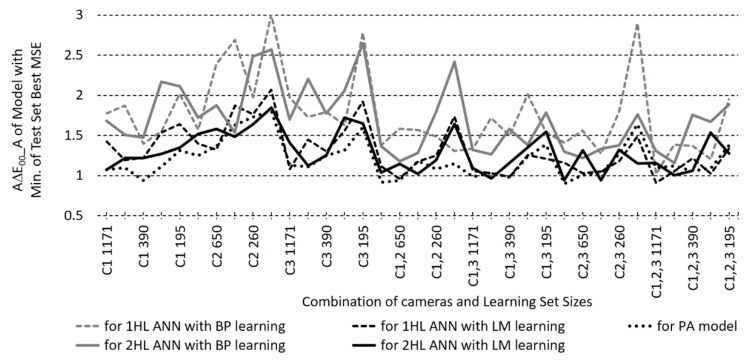
Comparison of test set average CIEDE2000 colour differences with illuminant A (A∆E*_00__A) for ANN and PA models which exert the best MSE of the test set, according to one and two hidden layer ANNs (1/2HL) modelled with BP or LM learning algorithm and for the polynomial approximation models (PA).

When the other performance indicators, i.e., GoFC and ∆E*_00_ for different illuminants, are also compared for the models with the smallest MSE, a similar performance ratio would be expected between the ANN and PA models. However, AGoFC and A∆E*_00_ performances of PA models appear to be better than all BP- and many LM-trained ANN models, not only with one but also with two and three cameras. The comparison of A∆E*_00_ for illuminant A of ANN and PA models exerting the best MSE is shown in Figure 9.

**Figure 10:**
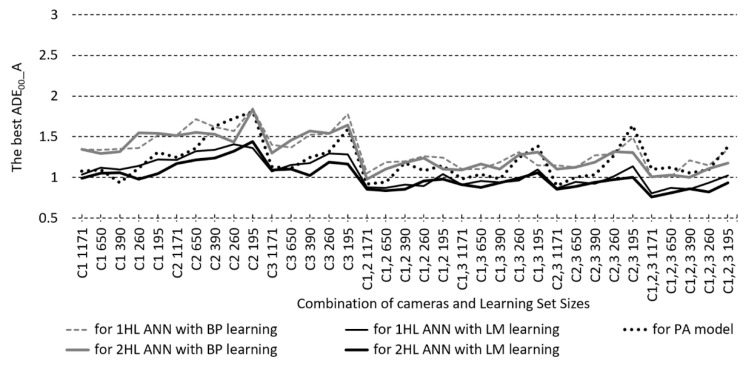
Comparison of the best test set average CIEDE2000 colour differences with illuminant A (A∆E*_00__A) for ANN and PA models, according to one and two hidden layer ANNs (1/2HL) modelled with BP or LM learning algorithm and for the polynomial approximation models (PA).

Next, the best AGoFC and A∆E*_00_ performances, which do not necessarily belong to the model with the smallest MSE, are searched out (through all HL1/HL2 NoN) at each camera-combination/LS size/learning algorithm combination of the ANN models and compared to the best PA model. These performances are found to be slightly worse in the case of BP models or overlap with the performance of PA models, whereas in the case of LM-trained ANN models, the best AGoFC and A∆E*_00_ surpass these performances of PA models. The comparison of the best A∆E*_00_ for illuminant A of ANN and PA models is shown in Figure 10.

### 4.6. A Profiling Approach to Search for the Best ANN Models

A profiling approach is presented for an efficient search of the best ANN models according to the selected combination of cameras, learning algorithm, and the preferred performance criterion.

In the previous comparison, it has been shown that for the selected combination of camera-combinations, LS size, and learning algorithm, when searching through all HL1 and HL2 NoN in the collection of ANN models with the best MSE, it is possible to find those models that are comparable or surpass the PA models for the observed performance, e.g., AGoFC or A∆E*_00_ for the selected illumination.

As presented in our previous study [15], searching for the best ANN model is time-consuming due to the nature of neural network modelling. With initial random values of the weights of connections between neurons, the model can result in a local minimum of the criterion function. Therefore, several iterations are needed to find more efficient ANN models for RS reconstruction.

Since the efficiency of the ANN model depends on all its hyper-parameters for a particular camera-combination/LS size/learning algorithm configuration, it makes sense to start the search for an efficient model at an appropriate point—the search centre, which is defined by the number of neurons in each HL—and then search its neighbourhood within a selected width (±9, 6 or 3 NoN). A broader search area presumably increases the probability of successfully finding more efficient ANN models, while a narrower one is less time-consuming.

So far, the calculated performance (MSE, AGoFC, or A∆E*_00_) has only been presented for the test set part of LS. When forming the LS, a part of the samples is always omitted, i.e., the independent-set, which increases as the LS size decreases (i.e., from 1171 to 650, …, to 195). The samples in the independent set do not change within the selected size of LS, in contrast to the test set, which changes with each iteration. When modelling ANNs, the performance of samples from the independent set, which is close to the test set performance, has always been calculated. For a broader performance assessment, in the profiling proposal presented below, the weighted sum (WS) of the sample test set and independent set performance is used.

In the following, a profiling proposal is presented to find effective ANN models for RS reconstruction, where the chosen performance criterion is the average colour distance A∆E*_00_ for standard light source A. Yang, Ming, and Yu [33] proposed seven quality classes (Hardly, Slight, Noticeable, Appreciable, Much, Very much, and Strongly Perceptible Colour Difference) to evaluate the colour differences ∆E*_00_. At the border between the “slight” and “noticeable” classes, there is a transition from a negligible colour difference to a colour difference perceptible to the human eye. For a presentation where a larger value should mean better performance, the differences between the value of “slight”–”noticeable” perception margin (hereafter SNPM) and the average weighted sum (hereafter AWS) of the observed performance were calculated.

**Figure 11:**
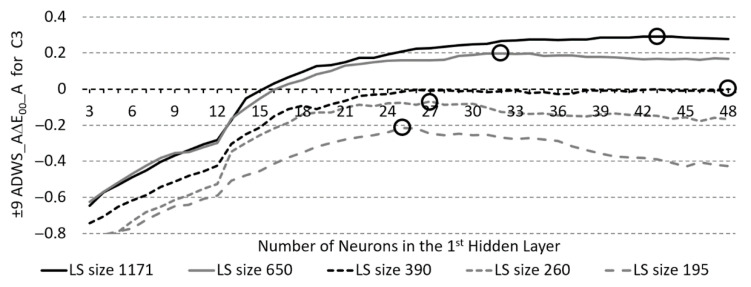
*Average* of ±9 number of neurons (NoN) neighbourhood of *Difference* between “slight”–”noticeable” perception margin and the *Weighted sum* of average CIEDE2000 colour differences with illuminant A (±9 ADWS_A∆E*_00__A), for the third single camera (C3 = GH4), and LM learning algorithm, with marked maximum values, according to different learning set (LS) sizes.

Profiling at the selected camera-combination and learning algorithm would be possible in several ways based on the calculated performance measures (MSE, AGoFC, A∆E*_00_) through the observed range of model parameters, e.g., by polynomial approximation or by moving average. In our profiling approach, the latter was used, where the moving average width was equal to the selected width of the search neighbourhood. The maximum of the curve thus obtained is the search centre, around which greater success in finding the best models can be expected. The search centre is, therefore, the NoN in the HL, around which, in the selected width of the neighbourhood, we are looking for ANNs, that, in the presented example, enable the smallest possible A∆E*_00_ for the reconstruction of RS. As already said, when choosing the width of the area, the relationship between a higher probability and higher speed of finding more successful models is also determined. Therefore, profiling was performed in three neighbourhood widths around the search centre, namely, ±9, 6, and 3 NoN, each time at five LS sizes. The *Average* of ±9 NoN neighbourhood of the *Difference* between SNPM and the *Weighted Sum* of A∆E*_00_ for illuminant A (abbreviated as ADWS_A∆E*_00_) through all observed learning set sizes, for the third single camera combination (C3 = GH4) and LM learning algorithm, with encircled maximum values is shown in Figure 11. In the case of ANN models with two HLs, with the selected camera-combination/learning algorithm configuration, we also get search centre profiling for HL2 NoN depending on the neighbourhood width and the LS size.

When the ANN profile is determined for the selected combination of camera-combination, learning algorithm, and performance criterion (e.g., A∆E*_00_), the NoN for the search centre can be read for the selected LS size and search neighbourhood width. To find the best ANN model for RS reconstruction, in the presented example, the search centre would be read, around which the best models in terms of the smallest MSE would be found in the selected neighbourhood (±NoN in the HL), and from these, the model with the smallest weighted average colour distance for test set and independent set.

**Figure 12:**
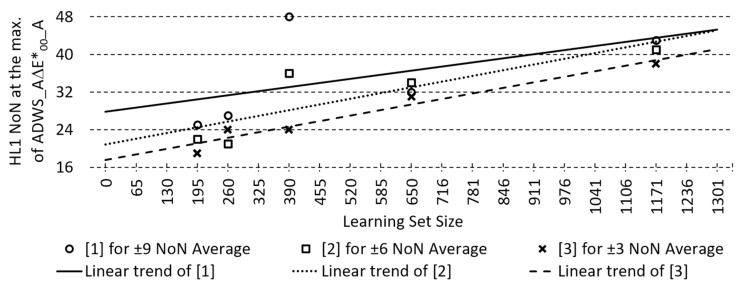
Number of neurons in the 1st hidden layer (HL1 NoN) at the max. of *Averaged Differences* between *“slight”–”noticeable” perception margin* and the *Weighted Sum* of *Average* CIEDE2000 colour differences with *illuminant A* (ADWS_A∆E*_00__A) at the observed learning set sizes and their linear approximations for GH4 camera (C3) according to the different *averaging interval* widths.

Due to the possibility of determining the search centre even with different LS sizes, a linear approximation was made for each profile, where the percentage of the best models found decreases, but the search centres determined in this way can still help guide the search for successful ANNs. Profiling the third camera (GH4) for determining the search centre for HL1 NoN in the search for the most successful LM-trained ANNs under the criterion of the smallest A∆E*_00_ is shown in Figure 12.

The performance evaluation of 1HL and 2HL ANN profiling for BP and LM learning algorithm and one, two, and three cameras in terms of the percentage of the top four models found and the percentage that at least one of the top four models is found is shown in Table 2.

The percentage of the best values found for 1HL ANNs increases with an increase in the number of cameras and with a wider search neighbourhood. For 2HL ANNs, the percentage increases with a wider search neighbourhood but varies according to the choice of learning algorithm, HL1/HL2 and number of cameras. For BP and LM learning algorithms at HL1, the percentage is highest at one, slightly lower at three, and lowest at two cameras. For BP learning algorithm at HL2, the percentage is very similar for one and three cameras and slightly lower for two. For LM and HL2, the percentage is also very similar for one and three but slightly higher for the two cameras. On average, linearisation reduces the percentage of best values found by 24% and, in only 9% of cases, by more than 50%.

At least one of the top four models is found almost everywhere. For 1HL BP and LM learning algorithms, this percentage is 100%, and for linearisation, it is on average 95%. For 2HL BP and LM, the average percentage for *HL1* is above 97%, and for linearisation, it is 88% for BP and 70% for LM. For 2HL BP and LM learning algorithms, the average percentage for *HL2* is above 98%, and for linearisation, it is 93% for BP and 82% for LM.

## 5. Discussion

The presented method of reconstructing the reflectance spectrum using information from reference samples captured in parallel with the photographed object has some limitations regarding camera settings and illumination, but these are not difficult to overcome:

- The capture mode of the camera should be set to manual mode and should not be changed during the time interval between the capture of the object and the capture of the colour reference chart.
- The illumination should be uniform over the area of the object and the reference chart. Uneven illumination, shadows, and reflections are undesirable.
- If possible, a reference colour chart of practical size (not too large) should be taken at the same time as the object of interest (e.g., a drawing or a painting). Otherwise, care should be taken to ensure constant illumination over the time interval of one capture after the other, which is particularly important when taking photographs in daylight.
- A flatbed scanner can be used to capture small flat objects. In this case, the scanner’s auto-correction options should be switched off during the interval of scanning the object and the reference chart in sequence.

When using multiple cameras to capture the object and reference colour chart, it is also necessary to ensure the registration of all captured images, which would be essential for practical implementation. Image registration is a broad topic that goes beyond the purpose of our experiment, in which the capture of the same coloured “pixels” by different cameras was ensured by successively capturing the same homogeneous area of a coloured patches under invariant illumination.

*The first comparison* (see the Results section) shows a better mean and best MSE performance of the two-layer ANNs with smaller NoN in the first layer than in the second one for almost all camera-combinations/LS size combinations and using both learning algorithms (Figure 4). The only exceptions are point overlapping curves of the mean MSEs for LM-trained ANNs using all three cameras and large-sized LS, but the curves of the best MSEs are well apart, and another point overlapping of the best MSEs for BP-trained ANNs using two cameras and the largest LS (not depicted), where the curves of the mean MSEs are well apart. With more ANN modelling repetitions, these overlappings would very likely diminish. For all the camera-combinations/LS size combinations, the BP- and LM-trained 2HL ANNs show a higher efficiency of RS reconstruction performance when utilising smaller NoN in HL1 than in HL2, which confirms our first hypothesis. This, in turn, means that for our fully connected ANN architecture with a few inputs (RGB responses) and many outputs (reflectance spectrum), less than half of all possible combinations need to be tested to find the ANN with the best performance. Even better, for a selected HL1 NoN, the best HL2 NoN only needs to be searched in the range from that HL1 NoN upwards. For 2HL ANNs, therefore, we can consider HL1 NoN as a more significant hyperparameter.

In *the second comparison*, the difference in MSE performance of the 1HL and 2HL ANNs with the same NoN in the first HL was compared for all camera-combinations/LS size combinations and for both—BP and LM—learning algorithms. As the number of calculated differences in this experiment is very big, their averages (AoND) through all HL1 NoN (for each camera-combination/LS size combination) were calculated for summary comparison. The BP-trained 2HL ANN models are somehow better than 1HL ANNs with the same NoN in HL1, but only in the search for the best MSE performance. On average, the 1HL ANN models are comparable to, if not even better than, 2HL ANN BP-trained models, especially if the 1HL vs 2HL ANN model learning times are to be considered. On the other hand, when examining the LM-trained 2HL ANN models, we can conclude that 2HL ANNs perform significantly better than 1HL ANN models with the same NoN in HL1, and show noticeably better average performance, both through the AoND for the best and mean MSEs. By focusing solely on 1HL and 2HL ANNs with the same NoN in HL1 and using the LM algorithm, the RS reconstruction performance is better using 2HL ANNs, which confirms our second hypothesis.

*The third comparison*—studying the change in performance when LS size decreases for the selected fixed number of cameras—shows that MSE and A∆E*_00_ (for all observed illuminations) for both ANN and PA RS reconstruction methods change only slightly for largest to medium LS sizes (1171 to 390), but increase for smaller LS sizes, with more rapid change manifested for the PA method. The AGoFC does not change or increases merely a little for ANN methods by reducing the LS size. For three-camera PA models, by reducing LS size, AGoFC improves insignificantly, whereas it degrades for one- and two-camera PA models. We can conclude that for both ANN and PA models with the selected number of cameras, reducing the size of the LS reduces performance in most cases, which supports our third hypothesis.

*The fourth comparison*—monitoring the change in performance when increasing the number of cameras at a constant LS size—shows that with an increasing number of cameras, both MSE and A∆E*_00_ (for all tested illuminants) in RS reconstruction with ANN models improve. In contrast, with PA models, MSE decreases slightly for two cameras compared to one but increases with three cameras, and A∆E*_00_ for three-camera models is mostly worse than for two-camera PA models. In ANN, the AGoFC in RS reconstruction only slightly improves with an increasing number of cameras, while in PA, except for the largest and smallest LS sizes, it improves a little with two cameras, then stagnates or decreases with three cameras.

We can conclude that the performance of ANN models increases the most when switching from one to two cameras and slightly less when switching to three. In PA models, switching from one to two cameras improves MSE, AGoFC, and A∆E*_00_ performances, while switching to three makes them worse. The reason for this may lie in the rapid increase in the number of parameters in PA models when increasing the number of independent variables. With a larger number of parameters, more data in the training set is needed for successful modelling. The number of parameters in PA models for RS reconstruction increases according to formula 4, where *N_SC_* is the number of spectral components (36 in our case), *N_in_* is the number of inputs to the system (3, 6, or 9 for RGB values of one, two, or three cameras), and *D_MVP_* is the degree of multivariate polynomials in the PA model.

(4) $$NP_{PA}=N_{SC}\left(\begin{array}{c} N_{in}+D_{MVP}\\ D_{MVP} \end{array}\right)$$

The number of parameters in PA models for one to three cameras and the use of multivariate polynomials of up to the seventh order is calculated in Table 3. The number of variants of 1/2/3 RGB PA models using polynomials of a certain degree according to the best and mean MSE is also shown. For the best MSE, considering weights, the average number of coefficients is calculated to be 1696, and for the mean MSE, 1232. Regardless of which of the two we take as the average value for finding optimal PA models, despite taking a large arbitrary search neighbourhood (e.g., −80% to +80% or 246 to 3052), there are only a few possible variants: for 1RGB PA models, multivariate polynomials of order between 2 and 6, for 2RGB models multivariate polynomial between 1 and 3, and for 3RGB PA models, polynomial of order 1 and 2. There is very little room for “fine-tuning” PA models.

The number of parameters of 1HL ANN models for RS reconstruction increases according to formula 5, and of 2HL ANN models according to formula 6, where *N_in_* is the number of inputs, *N_HL_*_1_ is the NoN in the 1st HL, *N_HL_*_2_ is the NoN in the 2nd HL, and *N_out_* is the number of outputs or NoN in the output layer.

(5) $$NP_{1HL\;ANN}=(N_{in}*N_{HL1}+N_{HL1})+(N_{out}*N_{HL1}+N_{outO})$$

(6) $$NP_{2HL\;ANN}=(N_{in}*N_{HL1}+N_{HL1})+\left(N_{HL2}*N_{HL1}+N_{HL2}\right)+(N_{out}*N_{HL2}+N_{out})$$

Calculations of the number of parameters for one to three cameras or three to nine inputs for 1HL ANN models are given in Table 4, and the number of parameters of 2HL ANN models is in Table 5. Due to the table size, only the variant for three cameras is presented. However, the number of parameters of the 2HL ANN models with one and two cameras does not differ significantly and, according to the table shown, decreases by 9*N_HL*1*_ with two cameras and by 18*N_HL*1*_ with one camera, which, e.g., for 3 HL1/3 HL2 NoN, means a reduction from 186 to 177 or 168, and for 48 HL1/48 HL2 NoN a change from 4596 to 4452 or 4308. For a comparison with PA models, the tables are shaded according to the range of search for the optimal number of coefficients in PA models.

In the case of 1HL and 2HL ANN models, there is an appreciable possibility of changing the number of parameters in small steps, which, in search of the optimal model for RS reconstruction, makes it much easier to adapt to the given *size of the learning set* with ANN models compared to PA models. This is particularly evident in the two- and three-camera models, where the range of the number of parameters of the PA models is very narrow and, in a realistic situation with a limited number of samples in the LS, better performance of the “fine-tuned” 2 and 3RGB ANN in comparison to PA models can therefore be expected, supporting our fourth hypothesis.

The fifth comparison—of the best MSEs between ANN and PA models—shows that for each combination of camera-combination/LS size, it is possible to find ANN models that are comparable to or outperform PA models, with LM learning being more efficient than BP learning. The comparison of AGoFC and A∆E*_00_ performance for these models with the smallest MSE does not speak in favour of ANNs, while comparing the best AGoFC and A∆E*_00_ for each camera-combination/LS size combination reveals that it is possible to find ANN models where the selected performance (e.g., A∆E*_00_ for a specific illumination) is comparable to, or even exceeds, the performance of PA models.

The findings of the fourth and fifth comparisons confirm that the RS reconstruction methods based on ANN and PA are comparable, although the ANN models have better fine-tuning capabilities. Given realistic constraints, such as the number of RGB input parameters and the LS size, it is thus possible to find ANN models whose performance outperforms PA models, confirming our fifth hypothesis.

For more effective searching for better ANN models, with the selected camera-combination, learning algorithm, and number of HLs, a method of profiling is proposed, which enables us, depending on the selected width of the search neighbourhood, to determine the centre of the search, i.e., NoN for HL1 (and HL2 for 2HL ANN) according to LS size. Searching for the best ANNs by scanning a wide range of possible HL1/HL2 NoN combinations is time-consuming, and by reducing this range using moving average profiling or its linearisation, which serves as an orientation for search centre determination even at different LS sizes, the search for effective ANN speeds up considerably. The evaluation of the profiling efficiency based on the percentage of the best four models found shows a higher efficiency for a wider search area. In the case of 1HL ANNs, the search efficiency also increases with an increasing number of cameras, which does not have a significant effect in the case of 2HL ANNs. The evaluation of profiling performance in terms of the probability of finding at least one of the top four models is slightly higher with a broader search neighbourhood but remains high across all selected search widths and all camera-combinations. The evaluation of profiling linearisation is, as expected, moderately lower. From the findings, it can be concluded that the primary profile with moving averaging ensures a higher success of finding efficient ANNs models, and the linearisation serves as an approximate orientation of where the search centre is located for LS sizes that are not included in the basic profiling.

## 6. Conclusions

The models considered in this study are generic, fully connected ANN models with a wide range of output values, as well as PA models with multivariate polynomials to calculate each of the output values.

In this context, models with a higher number of neurons in the second HL than in the first one were shown to be more efficient in computing 2HL ANN models for RS reconstruction, where there are more outputs than inputs. ANN models for RS reconstruction are also more flexible, as they allow more accurate tuning of the number of parameters according to the available size of the training set and, thus, better absorption of the information contained in the training set compared to PA models.

Are ANN models with two HLs more effective than those with one in reconstructing RS? This is certainly the case when comparing 1HL and 2HL models with the same number of NoNs in the first HL, but also otherwise, since 2HL ANNs modelled with the LM algorithm outperform 1HL models, both in the comparison where the size of the training set changes when the number of cameras (number of inputs) is kept constant, and in the comparison where the reverse is true. The same is true when comparing the performance of the best models found over all combinations of cameras and training set sizes, although here, the difference between 1HL and 2HL model performance is sometimes very small.

It was found that the improvement of the 2HL models with respect to the performance of the 1HL models is noticeable but not significant. Given the time complexity of modelling 2HL models, it is necessary to consider the time–cost–benefit ratio of modelling 2HL ANN models before designing a real-world application. Therefore, for a more prosperous and faster search, profiling of ANN models with a specific choice of camera configuration, learning algorithm, and performance criterion were also proposed, which allows us to determine the search centre—the initial search coordinates, i.e., the number of neurons in the HL(s), depending on the size of the training set and the search extent (width), in order to find more efficient ANN models for reflectance spectra reconstruction.

## Figures and Tables

**Table 1:** Learning Sets Sizes.

Descriptive Size of LS	% of LS vs a Complete Set of 1301 Colour Patches	Num. of 1/2/3 RGB-Reflectance LS Pairs	Num. of Remaining Independent Set Pairs
very large	90	1171	130
large	50	650	651
medium	30	390	911
medium	20	260	1041
smaller	15	195	1106

**Table 2:** Evaluation of the profiling performance of 1/2HL ANN for BP/LM learning algorithm (LA) with 1, 2, and 3 cameras in the percentage of the first four best models found and in the percentage of finding at least one in four best models.

			% of HL1/HL2 NoN Found						% That at Least One					
			of the Four Best Models at						of the Top Four Models Is Found at					
Hidden Layer	ANN Model and LA	NoN Neigh-bourhood	Max. of Avg. for			Linear Approx. for			Max. of Avg. for			Linear Approx. for		
			All Combinations of			All Combinations of			All Combinations of			All Combinations of		
			1CAM	2CAM	3CAM	1CAM	2CAM	3CAM	1CAM	2CAM	3CAM	1CAM	2CAM	3CAM
HL1	1HL BP	±9	71.7	76.7	90.0	53.3	53.3	80.0	100.0	100.0	100.0	100.0	100.0	100.0
		±6	65.0	71.7	85.0	41.7	43.3	70.0	100.0	100.0	100.0	100.0	93.3	100.0
		±3	48.3	58.3	65.0	30.0	35.0	55.0	100.0	100.0	100.0	80.0	86.7	100.0
HL1	1HL LM	±9	83.3	75.0	85.0	73.3	66.7	80.0	100.0	100.0	100.0	100.0	100.0	100.0
		±6	63.3	63.3	70.0	53.3	55.0	75.0	100.0	100.0	100.0	100.0	93.3	100.0
		±3	50.0	56.7	65.0	20.0	36.7	60.0	100.0	100.0	100.0	66.7	93.3	100.0
HL1	2HL BP	±9	91.7	73.3	85.0	90.0	70.0	80.0	100.0	100.0	100.0	100.0	100.0	100.0
		±6	88.3	53.3	70.0	76.7	48.3	65.0	100.0	93.3	100.0	100.0	93.3	100.0
		±3	75.0	43.3	50.0	58.3	23.3	25.0	100.0	100.0	80.0	80.0	60.0	60.0
HL1	2HL LM	±9	85.0	60.0	75.0	48.3	26.7	45.0	100.0	100.0	100.0	73.3	80.0	80.0
		±6	80.0	55.0	60.0	45.0	21.7	30.0	100.0	93.3	100.0	80.0	80.0	80.0
		±3	63.3	41.7	45.0	40.0	25.0	5.0	100.0	93.3	100.0	73.3	60.0	20.0
HL2	2HL BP	±9	80.0	70.0	75.0	78.3	63.3	70.0	100.0	100.0	100.0	100.0	100.0	100.0
		±6	70.0	56.7	75.0	65.0	56.7	70.0	100.0	100.0	100.0	100.0	100.0	100.0
		±3	53.3	45.0	55.0	43.3	41.7	45.0	93.3	93.3	100.0	93.3	93.3	100.0
HL2	2HL LM	±9	70.0	81.7	75.0	71.7	63.3	70.0	100.0	100.0	100.0	100.0	100.0	100.0
		±6	60.0	73.3	65.0	56.7	56.7	55.0	100.0	93.3	100.0	100.0	100.0	100.0
		±3	50.0	51.7	50.0	38.3	41.7	20.0	100.0	93.3	100.0	93.3	93.3	60.0

**Table 3:** Number of 1/2/3 RGB PA model coefficients according to the degree of in-model multivariate polynomials and number of calculated best PA models with polynomials of a certain degree according to mean and best MSE.

				Number of the Best					
Degree of Multivariate	Num. of All			1RGB	2RGB	3RGB	1RGB	2RGB	3RGB
Polynomials	1RGB	2RGB	3RGB	PA Model Variants					
in PA Model	PA Model Coefficients			According to the Best MSE			According to the Mean MSE		
1	144	252	360	0	0	0	0	0	0
2	360	1008	1980	0	**8**	**5**	**8**	**14**	**5**
3	720	3024	7920	**5**	**7**	0	**5**	**1**	0
4	1260	7560	25,740	**6**	0	0	**2**	0	0
5	2016	16,632	72,072	**3**	0	0	0	0	0
6	3024	33,264	180,180	**1**	0	0	0	0	0
7	4320	61,776	411,840	0	0	0	0	0	0

**Table 4:** Number of coefficients for 1/2/3RGB 1HL ANN models in step 2 for the number of neurons in the first hidden layer.

HL1 NoN	3	5	7	9	11	13	15	17	19	21	23	25	27	29	31	33	35	37	39	41	43	45	47
3 inputs	156	234	312	390	468	546	624	702	780	858	936	1014	1092	1170	1248	1326	1404	1482	1560	1638	1716	1794	1872
6 inputs	168	252	336	420	504	588	672	756	840	924	1008	1092	1176	1260	1344	1428	1512	1596	1680	1764	1848	1932	2016
9 inputs	180	270	360	450	540	630	720	810	900	990	1080	1170	1260	1350	1440	1530	1620	1710	1800	1890	1980	2070	2160

**Table 5:** Number of coefficients for 3RGB 2HL ANN models in step 3 for the number of neurons in the 1st and 2nd hidden layer.

		Number of Neurons in the 2nd Hidden Layer															
		3	6	9	12	15	18	21	24	27	30	33	36	39	42	45	48
**Number of Neurons in the 1st hidden layer**	**3**	186	306	426	546	666	786	906	1026	1146	1266	1386	1506	1626	1746	1866	1986
	**6**	225	354	483	612	741	870	999	1128	1257	1386	1515	1644	1773	1902	2031	2160
	**9**	264	402	540	678	816	954	1092	1230	1368	1506	1644	1782	1920	2058	2196	2334
	**12**	303	450	597	744	891	1038	1185	1332	1479	1626	1773	1920	2067	2214	2361	2508
	**15**	342	498	654	810	966	1122	1278	1434	1590	1746	1902	2058	2214	2370	2526	2682
	**18**	381	546	711	876	1041	1206	1371	1536	1701	1866	2031	2196	2361	2526	2691	2856
	**21**	420	594	768	942	1116	1290	1464	1638	1812	1986	2160	2334	2508	2682	2856	3030
	**24**	459	642	825	1008	1191	1374	1557	1740	1923	2106	2289	2472	2655	2838	3021	3204
	**27**	498	690	882	1074	1266	1458	1650	1842	2034	2226	2418	2610	2802	2994	3186	3378
	**30**	537	738	939	1140	1341	1542	1743	1944	2145	2346	2547	2748	2949	3150	3351	3552
	**33**	576	786	996	1206	1416	1626	1836	2046	2256	2466	2676	2886	3096	3306	3516	3726
	**36**	615	834	1053	1272	1491	1710	1929	2148	2367	2586	2805	3024	3243	3462	3681	3900
	**39**	654	882	1110	1338	1566	1794	2022	2250	2478	2706	2934	3162	3390	3618	3846	4074
	**42**	693	930	1167	1404	1641	1878	2115	2352	2589	2826	3063	3300	3537	3774	4011	4248
	**45**	732	978	1224	1470	1716	1962	2208	2454	2700	2946	3192	3438	3684	3930	4176	4422
	**48**	771	1026	1281	1536	1791	2046	2301	2556	2811	3066	3321	3576	3831	4086	4341	4596

## Data Availability

Not applicable.
